# Interview Day Environment May Influence Applicant Selection of Emergency Medicine Residency Programs

**DOI:** 10.5811/westjem.2016.10.31245

**Published:** 2016-11-15

**Authors:** Jason Lewis, Nicole Dubosh, Carlo Rosen, David Schoenfeld, Jonathan Fisher, Edward Ullman

**Affiliations:** * Beth Israel Deaconess Medical Center, Department of Emergency Medicine, Boston, Massachusetts; † Maricopa Medical Center, Department of Emergency Medicine, Phoenix, Arizona

## Abstract

**Introduction:**

The structure of the interview day affects applicant interactions with faculty and residents, which can influence the applicant’s rank list decision. We aimed to determine if there was a difference in matched residents between those interviewing on a day on which didactics were held and had increased resident and faculty presence (didactic day) versus an interview day with less availability for applicant interactions with residents and faculty (non-didactic day).

**Methods:**

This was a retrospective study reviewing interview dates of matched residents from 2009–2015.

**Results:**

Forty-two (61.8%) matched residents interviewed on a didactic day with increased faculty and resident presence versus 26 (38.2%) on a non-didactic interview day with less availability for applicant interactions (p = 0.04).

**Conclusion:**

There is an association between interviewing on a didactic day with increased faculty and resident presence and matching in our program.

## INTRODUCTION

Over the past five years the number of medical students applying to emergency medicine (EM) as well as the total number of EM residency positions has increased.^1^ Furthermore, the average number of ranked programs by residents matching in EM through the National Residency Matching Program now exceeds 11,^2^ leading to more interviews per applicant. Given the increased competition for candidates, a better understanding of the factors that influence applicants is important. Previous studies have shown that applicants frequently view the happiness of residents, program personality, ability to interact with residents, enthusiasm of the faculty, geographic location and interview day experience as most important when selecting a residency program both in EM and other specialties.^3^–^11^ Interactions with current residents and EM faculty may improve the prospective applicants’ understanding of the program. What is less evident, however, is whether the specific structure of an interview day influences student rank list.

Residency programs typically offer multiple interview days per week during the interview season. This may result in two distinct interview days. Often one day coincides with residency didactics and offers increased availability for interactions with residents and faculty members. In contrast, the second interview date may occur when there are no formal lectures and less availability for interaction with residency members. The goal of this study was to determine if there was a difference in matched residents between those interviewing on a day with increased resident and faculty presence versus an interview day with less availability for applicant interactions.

## METHODS

This was a retrospective study performed at a tertiary medical referral center in Boston, Massachusetts, that is home to a three-year academic emergency medicine residency program. The interview date sheets from the 2009–2015 match years were reviewed by three of the study’s authors and confirmed three subsequent times for accuracy. During this time period, residency interviews were offered twice a week. One of these days, referred to as “didactic day,” is when resident didactic lectures, departmental morbidity and mortality (M&M) conference, and protected faculty administrative time occur. The majority of the department core faculty and residents has protected time on these days; they are physically present in the EM administrative suite where the interview day takes place and there is an increased presence of residents and faculty at the interview day lunch. On the second interview day, “non-didactic day,” residents and the majority of faculty have no formal administrative or educational activities scheduled. While there is still a resident lunch organized on non-didactic days, often fewer faculty and residents are present.

The three reviewers extracted the following information from each interview data sheet: gender, whether they interviewed on a “didactic” versus “non-didactic” day, whether or not they matched at our residency program, United State Medical Licensing Examination (USMLE) Step 1 score, and applicant competitive score, which is an aggregate measure incorporating Step 1, letters of recommendation, clerkship grades and application materials used to assess whether an interview should be granted. We excluded matched applicants who completed a clerkship or rotation at the program as they had extensive exposure to the residency. Proportions and Fisher exact tests were calculated using JMP 12 pro (Cary, NC). We used a Wilcoxon Rank Sum for USMLE and applicant scores as these were non-normally distributed data. This study was reviewed by the institutional review board at our institution and was determined to be exempt from further review.

## RESULTS

From 2009–2015, 1,029 residency applicants were interviewed during the regular interview season. Fifteen applicants met exclusion criteria. This left a total of 1,013 for analysis. There was no difference in the distribution of applicants by interview day or gender (Table). Applicants who interviewed on a didactic day had a 1.69 increased odds of matching (p=0.04) (Table). There was no difference in applicant or USMLE Step 1 score for residents matched on a didactic versus non-didactic Day (Table).

## DISCUSSION

As the number of applicants continues to increase, programs have adjusted the total number of student interviews. To accommodate this increase, we created multiple interview days at our program. This study shows an association between “didactic day” and applicant matching in our program. There was no difference between the two groups with regard to variables such as USMLE Step 1 or applicant competitive scores. While previous studies have shown that a variety of subjective factors influence an applicant’s decision to rank a residency program highly, this is the first study to our knowledge looking at the influence of two distinct interview dates on residents matched in a program.

Prospective residents gather information on residency programs through multiple sources, including online forums, websites and word of mouth.^12^,^13^ The brief time spent on site during the interview day, however, is integral to their decision.^7^ While there is evidence that specific questions asked during an interview can influence an applicant’s decision,^14^ there are no studies about the specific structure of the interview day and influence on rank list. Two general graduate medical education (GME) residency program studies show that the most commonly cited factors that applicants weighed in their ranking were residency work environment gleaned from quality time with the program director, faculty and chair and informal interaction with residents and the relationship between faculty and residents within the program.^7^, ^8^ Our didactic days include M&M conference during which residents and faculty interact candidly in an educational setting. This experience provides applicants with a better understanding of the faculty-resident relationship as well as the teaching skills of the faculty. Furthermore, the increased presence of faculty and residents during the day provides more opportunity for informal conversations and an improved understanding of the general feel of the residency program. In contrast, the non-didactic days typically have fewer faculty and residents in attendance and the applicants are not exposed to the educational conference, which may influence the applicants’ perception of the program.

EM residency-specific studies have found similar factors of importance in applicants formulating their rank list as the two GME studies.^3^,^4^,^5^,^9^ Geographic location is frequently cited as one of the most important deciding factors, but this is out of the program’s control. However, factors that programs can influence include overall happiness of residents, faculty enthusiasm, and interview day.^3^,^4^,^5^,^9^ Similar factors in radiation oncology and radiology residencies have been shown to affect applicant rank list.^6^,^10^ DeIorio et al. argue that the experience during the interview day influences the applicant’s perception of how happy the residents seemed, program personality, and faculty enthusiasm.^9^ Likewise, Love et al. suggest that applicants become increasingly more sophisticated about the choices and their own personal priorities with respect to selecting a program over time, which may be influenced by interviewing and communicating with other applicants, residents and faculty.^5^ With a greater number of faculty members present and engaging in M&M on didactic days, applicants are likely able to more fully appreciate faculty involvement in departmental activities. Furthermore, the increased number of residents present may provide a broader number of applicant-resident interactions. While both of our interview dates share a common “night out” with the residents, which have been shown to be important for applicants,^11^ there is an increased presence of residents on didactic days. This combined with experiencing the faculty-resident interaction can provide greater insight into the program’s personality, which may be influential in determining the applicants rank list.

Our results show that increased exposure to departmental activities and increased availability of faculty and resident interactions may positively influence the applicant match. The interview-day experience and interaction with faculty and residents may be a significant modifiable factor of the overall structure of the interview day.

## LIMITATIONS

There are several limitations to our study. We had a relatively small sample size at only a single site. While we did account for previous rotators in our ED who matched into our program, we were unable to account for other potential confounding variables such as home institution, couples matching, city preference, and family considerations. Additionally, we could not account for applicants who did not match into our program and the reason for ranking other programs more highly. Finally, we do not have an objective measurement of the number of faculty and residents in attendance on any given interview day.

## CONCLUSION

Our study found that the majority of residents who matched into our program interviewed on a “didactic day.” The greater presence of faculty and residents and increased availability for individual interactions among the applicants may provide better insight into the program and may prove beneficial for recruiting applicants. Larger and potentially multicenter studies would be needed to explore the full impact of increased resident and faculty presence and exposure to didactics as part of the interview day.

## Figures and Tables

**Table:** Interviewed and matched applicants with applicant score and USMLE Step 1 score by interview day from the 2009–2015 match.

	Didactic day	Non-didactic day	P-value
Included applicants	504 (49.8%)	509 (50.2%)	--
Male	296 (58.7%)	307 (60.3%)	0.61
Applicants matched at our program	42 (61.8%)	26 (38.2%)	0.04
Median applicant score	35	34	0.34
Median USMLE Step 1 score	239.5	240	0.95

*USMLE*, United States Medical Licensing Examination.
